# Are both the knees of the same size? Analysis of component asymmetry in 289 bilateral knee arthroplasties

**DOI:** 10.4103/0019-5413.80044

**Published:** 2011

**Authors:** Venkata Gurava Reddy, Aditya Krishna Mootha, Chiranjeevi Thayi, Pareen Kantesaria, Ramireddy Vinodh Kumar, Divakar Reddy

**Affiliations:** Department of Orthopedics, Sunshine Hospitals, Secunderabad, Andhra Pradesh, India

**Keywords:** Component asymmetry, sizing mismatch, bilateral total knee arthroplasty

## Abstract

**Background:**

Variations in the anatomy of knee are well described, however the true incidence of component asymmetry in bilateral total knee arthroplsties is rarely reported. Incidence of component asymmetry in bilateral total knee arthroplasties (TKA) was retrospectively analysed in 289 cruciate retaining total knee arthroplasties.

**Materials and Methods:**

Medical records of these 289 patients were evaluated for the incidence of asymmetry of either femoral or tibial components. Clinical outcomes were compared between the cases of asymetrical components to that of symmetrical components.

**Results:**

Incidence of femoral component asymmetry was found to be 9.2% and tibial component asymmetry to be 8.7%. Of 289 cases, TKA 178 were done in a single day (group A), while 111 were done at 2- to 3-day intervals (group B). Asymmetric and symmetric knees were equally distributed among both groups, male and female patients in both groups, and the incidence of component asymmetry was similar between all four different implants – Optetrak-CR (Exactech, Gainesville, FL, USA), Nexgen-CR (Zimmer, Warsaw, IN, USA), PFC-Sigma CR (DePuy, Warsaw, IN, USA), Genesis II CR (Smith and Nephew, Memphis, TN, USA) we used. The pre- and postoperative range of motion and pre- and postoperative knee society scores were compared between the symmetric and asymmetric cases in both the groups and the difference was found to be insignificant.

**Conclusion:**

We conclude that incidence of component asymmetry in bilateral total knee arthroplasty is around 9 % and independent sizing of both knees during bilateral arthoplasty is recommended rather than simply relying on the contralateral knee measurements.

## INTRODUCTION

Total knee arthroplasty (TKA) is commonly indicated in osteoarthritis, rheumatoid arthritis, other inflammatory arthritis, and posttraumatic arthritis. Although the majority of patients with arthritis of the knee undergo unilateral TKA, not infrequently do patients undergo bilateral TKA. One of the several factors which affect the final outcome is proper sizing of the components.^1^

The importance of correct sizing of components for TKA for an optimal function and long-term results has been stressed in many reports.^2^,^3^

An improper sizing of the femoral component can lead to a flexion–extension gap mismatch. A large-sized femoral component can lead to the loss of the flexion space thus leading to the postoperative loss of flexion and also overstuffing of the patellofemoral joint. 1 A undersized femoral component can lead to flexion instability. An improperly sized tibial component can lead to posterolateral overhang of the implant that can impinge on the popliteus and posterolateral corner. In patients undergoing bilateral total knee arthroplasty, it is important to size both the knees independently as both the knees may not be of same size all the time in a given patient. There are studies where component asymmetry in bilateral TKA has been analyzed,^4^,^5^ but to our knowledge, there have been no similar studies in the Indian population so far.

In the current study, we did a retrospective analysis of all the patients who underwent bilateral TKA with the hypothesis that both sides in these patients may not be symmetrical in all. We analyzed the incidence of asymmetry of femoral and tibial components among bilateral knee arthroplasty cases and evaluated the outcome of those cases.

## MATERIALS AND METHODS

We analyzed retrospectively all the bilateral knee arthroplasties done at our institute from April 2009 to May 2010 after getting the clearance from our Institutional ethical committee (IEC). During this period, a total of 324 bilateral TKAs were done either in a single day simultaneously or at an interval of 2–3 days (single hospital admission). We included only those cases (a) where prostheses of the same model and manufacturer were used in both the knees, (b) who had data regarding the implant details available, (c) who had a minimal follow-up of 6 months, and (d) whose preoperative and follow-up knee society scores^6^ were available. Cases excluded were (a) those where arthroplasty was done at different admissions for each knee, (b) those who had any postoperative complication in either of the knees such as deep vein thrombosis and infection, which might affect the final outcome, (c) those lost to follow-up. However, a detailed analysis of intraoperative notes of these patients showed that in five of these patients, a tibial or femoral cut was increased by 2 mm in one of the knees for the sake of soft tissue balancing. As this would have affected the component size, these five cases were also excluded.

A total of 289 patients out of 324 operated during this period met our inclusion and exclusion criteria.

The preoperative evaluation of the patients included detailed history, clinical assessment, diagnosis, anteroposterior and lateral X-rays, and routine hematological workup. In patients older than 70 years of age and those with associated comorbid conditions which might predispose them to moderate to high risk for adverse events, we did staged arthroplasty at an interval of 2–3 days under the same hospital admission. We considered this group of patients different for the purpose of statistical analysis as their mean age and scores differed as compared to those who had got the surgery done on the same day.

All the cases were operated by either of the two senior surgeons (GRA, CT). However, in a given patient, both the sides were operated by only one surgeon. In all the cases, cruciate-retaining arthroplasty was done. The implants used included Optetrak-CR (Exactech, Gainesville, FL, USA) (n=92), Nexgen-CR (Zimmer, Warsaw, IN, USA) (n=71), PFC-Sigma CR (DePuy, Warsaw, IN, USA) (n=67), and Genesis II CR (Smith and Nephew, Memphis, TN, USA) (n=59). All the cases were operated under the tourniquet by the medial parapatellar approach. Both the surgeons followed similar technique of sizing the components and if the femur appeared to be in between the different sizes, we preferred the higher size to avoid the complication of anterior femoral notching. In all the cases, femoral sizing was done according to the anterior referencing technique.^3^ All the tibia were sized in such a way that there occurred no overhang of the implant in either mediolateral or anteroposterior plane and the implant was in correct rotation. Both the surgeons did not resurface patella.

Postoperative protocols were same for all the patients which included injectable antibiotics for 3 days, DVT prophylaxis till the patients became ambulant and a fixed physiotherapy protocol which included full weight bearing walking by post operative day 1 or as soon as the pain allows, Bed side knee bending by post operative day 2. Follow-up evaluation was done at 3 weeks, 6 weeks, 3 months, and 6 months which included range of motion and clinical knee society score. Radiological evaluation during follow up included standing AP and lateral views at 3 weeks follow up to look for overall alignment and component placement.

All the cases were broadly divided into two groups: group A where both knees were operated simultaneously in a single day and group B where a staged arthroplasty was done at 2- to 3-day interval. Though the asymmetry of the components has little relation to whether surgery is done on same day or 3 days apart, the mean age, preoperative data like range of motion and knee society score differed among these 2 groups [Table 1]. The groups were analyzed for component asymmetry between the two knees. All the comparative analysis was done between those cases which had component asymmetry and those where the bilateral components were symmetrical, individually in both the groups. All the statistical analysis was done using SPSS 12.0 software and an unpaired *t* test was used for the comparison of numerical data like comparison of range of motion (ROM) and Knee Society score (KSS) among various groups. For all purposes, a *P* value <0.05 was considered to be statistically significant.

**Table 1:** Comparison of pre operative data between group A and group B patients

	Group A (n=178)	Group B (n=111)	*P* value
Pre operative ROM	111.09±6.97	104.00±10.48	0.00
Pre operative KSS	47.35±4.26	39.89±1.90	0.00

ROM: Range of motion expressed in degrees; KSS: Knee Society score; SD: Standard deviation

## RESULTS

The diagnosis was primary osteoarthrosis in 224 cases and rheumatoid arthritis in 65 cases. There were 178 cases where arthroplasty was done on the same day (group A) and 111 cases where a staged arthroplasty was done at 2- to 3-day interval (group B). The mean age of the patients in group A was 56.2 years (50 – 62 yrs) while it was 70.4 years (65-82 yrs) in group B. The male-to-female ratio was 78:100 in group A, while it was 50:61 in group B.

The femoral asymmetry occurred in a total of 27 cases (9.3%), while the incidence of tibial component asymmetry was seen in 25 cases (8.6%). The male–female difference in these cases was found to be insignificant for both the femoral (*P* value 0.26) and tibial component (*P* value 0.43). The incidence of asymmetry among the four different implants used is described in detail [Table 2]. The incidence of femoral component asymmetry in group A was 8.9% (16 cases) while in group B it was found to be 9.9% (11 cases) and the difference was statistically insignificant (*P* value 0.12). Similarly, the incidence of tibial component asymmetry was 8.4% (15 cases) and 9.0% (10 cases) in group A and B, correspondingly. The difference was statistically insignificant (*P* value 0.34). Of the 27 cases of femoral asymmetry, in 25 cases the asymmetry was by 1 size, while in 2 cases it was 2 sizes apart. All these 2-size apart cases were osteoarthritis cases. In 20 cases the right side was larger than the left side, while in the other 7 cases the left side was larger than the right side. Similarly, of 25 cases of tibial asymmetry, 24 cases were 1 size apart while in 1 case it was 2 sizes apart. In 17 cases, the right side was larger than the left side, while in other 8 cases, the left side was larger than the right side.

**Table 2:** Analysis of component asymmetry among the four different implants used

Implant	No. of cases (% of total cases)	Femoral asymmetry (%)	Tibial asymmetry (%)
PFC-Sigma (DePuy, Warsaw, IN, USA)	67 (23.2)	7 (10.4)	5 (7.46)
Nexgen (Zimmer, Warsaw, IN, USA)	71 (24.6)	6 (8.45)	7 (9.8)
Optetrak (Exactech, Gainesville, FL, USA)	92 (31.8)	8 (8.7)	8 (8.7)
Genesis-II (Smith and Nephew, Memphis, TN, USA)	59 (20.4)	6 (10.2)	4 (6.8)
Total	289	27 (9.3)	25 (8.6)

The mean pre operative ROM in group A was 111.09±6.97 while in group B it was 104.00±10.48. The mean pre operative KSS in group A was 47.35±4.26 compared to 39.89±1.90 of group B. Statistical analysis showed that the difference between group A and B for both the parameters is significant [Table 1]. The cases where the component asymmetry occurred were compared with those of symmetrical cases in relation to preoperative ROM, postoperative ROM, preoperative KSS, and postoperative KSS. Statistical analysis showed no significant difference between these cases in both groups [Tables 3 and 4].

**Table 3:** Comparative analysis of component asymmetrical and symmetrical cases in group A

	Group A					
	Femoral component			Tibial component		
	Asymmetry	Symmetry	*P* value	Asymmetry	Symmetry	*P* value
No. of cases	16	162		15	163	
Preop. ROM (mean ± SD)	110.9 ± 7.3	111.1 ± 6.9	0.92	110.7 ± 8.3	111.6 ± 7.5	0.66
Postop. ROM (mean ± SD)	120.6 ± 9.8	117.7 ± 10.2	0.28	123.7 ± 7.7	120.9 ± 10.3	0.32
Preop. KSS (mean ± SD)	48.4 ± 4.5	47.2 ± 4.2	0.28	47.2 ± 6.7	44.9 ± 5.9	0.15
Postop. KSS (mean ± SD)	88.7 ± 4.1	89.0 ± 7.2	0.83	88.9 ± 4.2	89.9 ± 3.9	0.36

ROM: Range of motion expressed in degrees; KSS: Knee Society score; SD: Standard deviation

**Table 4:** Comparative analysis of component asymmetrical and symmetrical cases in group B

	Group B					
	Femoral component			Tibial component		
	Asymmetry	Symmetry	*P* value	Asymmetry	Symmetry	*P* value
No. of cases	11	100		10	101	
Preop. ROM (mean ± SD)	102.7 ± 9.0	107.9 ± 9.6	0.09	98.0 ± 10.0	104.6 ± 10.4	0.06
Postop. ROM (mean ± SD)	110.0 ± 7.4	115.8 ± 9.8	0.06	107.0 ± 7.9	111.2 ± 6.9	0.07
Preop. KSS (mean ± SD)	45.9 ± 3.8	44.8 ± 5.9	0.55	41.0 ± 2.3	39.8 ± 1.8	0.06
Postop. KSS (mean ± SD)	85.9 ± 4.9	87.3 ± 9.0	0.60	82.9 ± 4.3	85.9 ± 5.2	0.07

ROM: Range of motion expressed in degrees; KSS: Knee Society score; SD: Standard deviation

## DISCUSSION

TKA continues to be one of the most effective orthopedic operative procedures and is considered to be extremely safe.

Anatomical variations in the size of the normal knee have been studied^7^ however, asymmetry of knees in bilateral arthroplasties is not much documented. In a review of 268 patients who underwent either simultaneous or staged bilateral TKA, Brown and Diduch^4^ reported asymmetry rates for femoral (6.7%), tibial (1.1%), and patellar components (0.3%). In a review of 253 patients who underwent simultaneous bilateral TKA, Capeci *et al*. reported the asymmetry rates of 8.7%, 6.7%, and 5.1% for femoral, tibial, and patellar components, respectively.^5^ Our study is the first to document in detail the component asymmetry analysis in the Indian population. In our current study, we found a slightly higher incidence of 9.3% for the femoral component and 8.6% for the tibial component. Asymmetry of the components was found with all the implant types we used. Component asymmetry was found in both the groups with a similar incidence whether the surgery was done on the same day or 2–3 days apart.

The importance of proper sizing of the components during TKA cannot be overemphasized. An improper sizing of the femoral component can lead to a flexion–extension gap mismatch. A large-sized femoral component can lead to the loss of the flexion space thus leading to the postoperative loss of flexion and also overstuffing of the patellofemoral joint. An undersized femoral component can lead to flexion instability. An improperly sized tibial component can lead to the posterolateral overhang of the implant that can impinge on the popliteus and posterolateral corner.

The strength of this study is that both surgeons performing the bilateral TKAs in this study independently sized components for each knee during the arthroplasties, demonstrating a difference in bony anatomy between right and left knees in a certain number of patients. The findings of similar postoperative knee scores and ROM provide support that proper component selection was achieved for each individual knee, whether the bilateral TKA was symmetrical or asymmetric. The fact that the patients included in the present analysis were all operated on a single admission thus reducing the bias adds to the strength of the study. The drawback of this study is this is a retrospective analysis, the radiological grading of the disease is not considered and true anthropometric measurements of the femur and tibia were not done either pre or per operatively, which would better document the actual difference of sizes in these knees.

However, a comparison of the postoperative results shows that the surgeons didn’t err as even the asymmetrical cases had similar results compared to symmetrical cases.

We conclude that the incidence of asymmetry of components in bilateral TKA is around 9% and hence we recommend that each knee must be sized independently, and solely relying on the contralateral knee measurements might lead to improper implantation. Further studies are needed to identify the risk factors for such situations.
